# The roles of health literacy and social support in the association between smartphone ownership and frailty in older adults: a moderated mediation model

**DOI:** 10.1186/s12889-024-18163-z

**Published:** 2024-04-17

**Authors:** Jinseon Yi, Ju Young Yoon, Chang Won Won, Miji Kim, Kyoung Suk Lee

**Affiliations:** 1 College of Nursing, Seoul National University, Seoul, Korea; 2 Research Institute of Nursing Science, Seoul National University, Seoul, Korea; 3 Center for Human-Caring Nurse Leaders for the Future by Brain Korea 21 (BK 21) Four Project, College of Nursing, Seoul National University, Seoul, Korea; 4 Department of Family Medicine, College of Medicine, Kyung Hee University, Seoul, Korea; 5 Department of Biomedical Science and Technology, College of Medicine, East-West Medical Research Institute, Kyung Hee University, Seoul, Korea

**Keywords:** Smartphone, Health literacy, Social support, Frailty, Digital health, Digital divide

## Abstract

**Background:**

Understanding the role of smartphones to promote the health status of older adults is important in the digital society. Little is known about the effects of having smartphones on physical frailty despite its positive effect on the well-being of older adults. This study aimed to explore the association between smartphone ownership and frailty in community-dwelling older adults and its underlying mechanism.

**Methods:**

We used data from the Korean Frailty and Aging Cohort Study and analyzed 2,469 older adults aged 72–86 years. Frailty, health literacy, and social support were assessed by Fried’s frailty phenotype, the Behavioral Risk Factor Surveillance System health literacy module, and the Enhancing Recovery in Coronary Heart Disease (ENRICHD) Social Support Instrument, respectively. The mediation model and moderated mediation model were estimated, where the mediator was health literacy and the moderator was social support, to explore the relationship between smartphone ownership and frailty.

**Results:**

Of our study participants, 58.9% owned smartphones, and 10.9% were classified as frail. Smartphone ownership was negatively associated with frailty (*β =* −0.623, *p <* 0.001*).* Health literacy mediated the relationship between smartphone ownership and frailty (*β* = −0.154, boot confidence interval [CI] = − 0.222, − 0.096), and social support moderated the mediation effect (*β =* −0.010, Boot CI = − 0.016, − 0.004).

**Conclusions:**

Owning smartphones among older adults could reduce the risk of frailty. Promoting health literacy and social support among older adults with smartphones would be effective to prevent frailty.

**Supplementary Information:**

The online version contains supplementary material available at 10.1186/s12889-024-18163-z.

## Background

A smartphone is a mobile phone that combines the functionalities of a computer, featuring a touchscreen interface, internet access, and an operating system capable of running applications [1]. The global penetration rate of smartphones reached 78.1% in 2020, dramatically increasing from 49.4% over 5 years [2]. A substantial rise was noticed even among older adults, who were initially trailing behind the younger generations in smartphone adoption. For instance, in the United States, the share of older adults aged ≥ 65 years who own smartphones increased from 30% in 2015 to 61% in 2021 [3]. Similarly, the proportion of smartphone users aged ≥ 60 years has shown remarkable growth, surging from 70% in 2018 to 90% in 2022 in Korea [4].

A digital divide among older individuals remains even with advancements in reducing the gaps in digital technology usage between generations. The 2022 Report on the Digital Divide in Korea presented that only 63.1% of individuals aged ≥ 70 years owned a smartphone, which is significantly lower compared to 93.6% among those in their 60s. Moreover, older adults who were males and had higher income levels exhibited higher rates of smartphone ownership and proficiency [5]. Potential factors cause the digital divide, such as low literacy, minority status, lack of interest or motivation to use technology, and limited access to technology, in addition to older age, female sex, and poverty [5, 6]. The advent of smartphones has resulted in significant changes in the healthcare environment, especially within the realm of digital healthcare [7]. Therefore, the digital divide, including gaps in smartphone ownership, is expected to contribute to inequalities in healthcare access and individual health outcomes [6, 8].

Previous studies have reported the positive influence of smartphone ownership on the well-being of older adults. Smartphone use in older adults was associated with higher life satisfaction, frequent social participation, and lower depression [9]. Furthermore, smartphone ownership has improved life satisfaction by lowering depressive symptoms [10]. Research investigating the effects of smartphone ownership on physical health remains lacking and the underlying mechanism of this association remains unknown although studies have documented its positive impact on the psychological health of older adults.

Frailty is a composite index that reflects the overall physical function of older adults. It is a state of increased vulnerability to stressors caused by aging-associated physiological changes [11]. Frailty is generally recognized as a significant adverse health outcome predictor in older adults, such as falls, hospitalization, and mortality [11]. Therefore, exploring the relationship between smartphone ownership and frailty can expand our understanding of the effect of smartphone ownership on the physical health of older adults.

Medical professionals or mass media, such as newspapers, radio, or television, are the traditional sources of health information. However, the Internet has now become the most popular and accessible source of health information, especially with the widespread smartphone availability [12]. Improved health literacy among smartphone owners should be recognized by ensuring their access to online health information and health-related applications although current evidence of health literacy has not mediated the relationship between smartphone ownership and frailty [13–15]. Additionally, higher health literacy levels are expected to yield positive health outcomes. Health literacy refers to the ability to find, understand, and use health information and services to make informed health-related decisions and actions [16]. Adequate health literacy is particularly important for older adults, because it empowers them to effectively manage chronic disease and complications, adopt healthy behavior, and utilize appropriate healthcare services [17]. Hence, this improved health outcomes in older adults, including enhanced physical function, reduced frailty, and decreased mortality [18–21].

All older adults were unlikely to experience an equal decrease in this risk through smartphone use although smartphone ownership among older adults has the potential to reduce the risk of frailty by improving their health literacy. This variability is influenced by social support. The lack of social networks and social support generally elevates the risk of frailty among older adults residing in the community [22]. Moreover, older adults often encounter age-related barriers that pose challenges to smartphone usage, including low computer literacy, decreased cognitive function, unfamiliar interfaces (e.g., small fonts, icons, scroll bars), and lack of motivation [23]. Therefore, the availability of social support can facilitate older adults’ new technology exploration and adoption [24, 25]. Additionally, social support can enhance the health literacy of older adults, as well as improve their self-efficacy and interest in technology while reducing technophobia [26, 27].

**Fig. 1:**
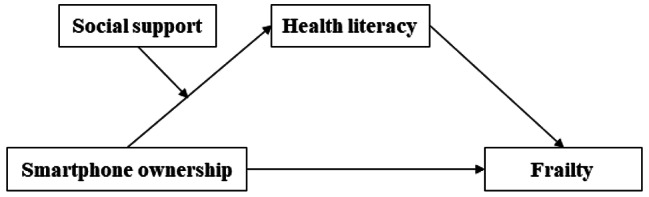
Hypothesized model of the relationship of study variables

The growing adoption of smartphones among older adults is expected to affect healthy and active aging promotion because of the significant potential of smartphones. However, the underlying mechanism of how smartphone ownership could reduce the risk of frailty remains unclear. The present study aims to investigate the mediating role of health literacy and the moderating role of social support in the relationship between smartphone ownership and frailty among community-dwelling older adults in Korea to address this research gap. Figure 1 presents the proposed model for the study.

We propose the following hypotheses based on the previous studies and their findings.

**Hypothesis 1**

Smartphone ownership and frailty will have a negative relationship among older adults.

**Hypothesis 2**

Health literacy will mediate the relationship between smartphone ownership and frailty.

**Hypothesis 3**

Higher social support levels will strengthen the mediating effect of health literacy in the relationship between smartphone ownership and frailty.

## Methods

### Data source and study participants

The Korean Frailty and Aging Cohort Study (KFACS) is a nationwide cohort study designed to assess the frailty status of older adults and its progression over time [28]. This cohort study has been conducted every 2 years, and our study used the data from the second wave (2018–2019) of KFACS, which was the most recent available. The third wave (2020–2021) and the fourth wave (2022–2023) have recently been conducted, but data had not been released when authors started the study.

Older adults aged 70–84 years were recruited from 10 sites across various regions in South Korea. The recruitment process ensured the representation of older adults in Korea by employing age and gender stratification. The inclusion criteria for participation were older adults currently residing in the community, with no plans of moving out within the next 2 years, no communication problems, and no dementia diagnosis [28]. This study selected 2,469 individuals after excluding cases with withdrawal (*n* = 3), those who didn’t complete questionnaires for the dependent variable (*n* = 502), health literacy (*n =* 14), social support (*n* = 19), and covariates (*n =* 7). We confirmed no gender difference between older adults included and excluded in this study (*χ*^*2*^ = 0.02, *p* = 0.902). However, the individuals who were excluded were on average 1.21 years older than those who were included (t = 6.59, *p* < 0.001).

### Measurement

#### Frailty

Frailty was measured using Fried’s frailty phenotype, which is a well-validated and widely used tool for frailty assessment in geriatric research [11]. This tool comprises five components. Unintended weight loss was an unintentional loss of > 4.5 kg in the past year [28]. Weakness was the lowest 20% of hand grip strength, adjusted for sex and body mass index (BMI) quartile based on KFACS baseline survey [29]. Exhaustion was a positive response to either of the following statements from the Center for Epidemiological Studies Depression scale: “I felt that everything was an effort” and “I could not get going” [30]. Slowness was measured by a usual gait speed of > 4 m, including an acceleration/deceleration phase of 1.5 m. Slowness was the lowest 20% of mean gait speed stratified by sex and height [28]. Physical activity was measured with the International Physical Activity Questionnaire. Energy consumption in a previous week was calculated as the product of metabolic equivalent scores [31]. Low physical activity was < 494.65 kcal/week for males and < 283.50 kcal/week for females [31]. Participants who exhibited three or more of the five components were classified as having frailty, while those who did not meet the criteria were categorized as non-frailty.

#### Smartphone ownership

Smartphone ownership was assessed through a questionnaire that inquired, “Do you have a mobile phone?” Participants were categorized into two groups based on the type of mobile phone they possessed: smartphone owners (those with smartphones) and non-smartphone owners (those with feature phones or without mobile phones).

#### Health literacy

Three items from the 2016 Behavioral Risk Factor Surveillance System questionnaires, developed by the United States Center for Disease Control and Prevention, were used to assess health literacy [32]. Participants were asked to respond to three questions inquiring about the difficulty level they experienced in finding health information and understanding oral and written health-related information. Each item was rated on a 4–point Likert scale, ranging from 1 (very difficult) to 4 (very easy). Response options, such as “do not know,” “refused,” and “not applicable or missing,” were given zero points. The total score ranged from 0 to 12, with a higher score indicating a higher health literacy level. The Cronbach’s alpha coefficient for this study was 0.78.

#### Social support

The Enhancing Recovery in Coronary Heart Disease (ENRICHD) Social Support Instrument (ESSI) measures social support [33]. The ESSI is widely employed in surveys conducted on community-dwelling older adults, such as the Establishing Populations for Epidemiological Studies of the Elderly (EPESE) and the Health and Retirement Study (HRS), and it had demonstrated good psychometric properties within the Korean older population [22]. The ESSI comprises six items that measure instrumental and emotional support, along with one item assessing structural support. Participants responded on a scale ranging from 1 (none of the time) to 5 (all of the time) for the six items, while the seventh item (marital status) was scored as 4 for “yes” and 2 for “no.” The total score ranges from 8 to 34, with higher scores indicating a greater social support level. Cronbach’s alpha coefficient was 0.86 in both the original and the present study, suggesting strong internal consistency [33].

#### Covariates

Covariates included age, gender, marital status, educational level, and monthly household income, as they have been identified as factors associated with both health literacy and frailty [34, 35]. Participants’ age was recorded as a continuous variable, ranging from 72 to 86 years. Marital status was dichotomized into two categories: married and others (never married, divorced, separated, and widowed). Educational level was classified as below elementary school, elementary school graduate, and over middle school graduate. Monthly household income was categorized into three groups: <1 million KRW, 1–2 million KRW, and > 2 million KRW, based on the ordinal scale used in the original survey.

### Ethical considerations

The KFACS operating committee reviewed the study design following their data provision policy. Data was provided without personally identifiable information. This study obtained an exemption from review by the Institutional Review Board of Seoul National University (IRB No. E2207/004 − 001) as this study used secondary data.

### Data analysis

Frequencies and percentages were used for categorical variables and means and standard deviations (SD) for continuous variables to describe the characteristics of study participants. Correlations among variables were examined with Pearson’s coefficients for intercontinuous-continuous variables, point-biserial coefficients for intercontinuous-binary variables, and phi coefficients for interbinary–binary variables. The mediation and moderated mediation models were examined using the PROCESS macro models 4 and 7, respectively, and the index of moderated mediation was estimated, which presents the association between the indirect effect and the putative moderator of that effect [36, 37]. All models were adjusted for covariates, including age, gender, marital status, educational level, and household income, and the bias-corrected 95% confidence interval (CI) were obtained using 5,000 bootstrapping resamples. The Johnson-Neyman technique was used to explore the conditional effect of smartphone ownership on health literacy across the range of social support. All the analyses were performed using STATA/IC version 14.0 (Stata Corp, College Station, TX) and IBM SPSS version 26.0 (IBM Corporation, Chicago, IL) with PROCESS macro version 4.0. All statistical significance was set at *p*-values of < 0.05.

## Results

### Participant characteristics

Table 1 shows the participant characteristics. The average age was 77.8 years (SD = 3.8), and the majority of them belonged to the 75–79 years category (41.7%). The majority of participants (66.0%) had a monthly income below 2 million KRW, which falls within the second lowest quintile of the average monthly household income for all Korean households in 2019 [38]. Among 2,469 participants, 269 (10.9%) were classified as frail, and 1,454 (58.9%) owned smartphones. Frail older adults were characterized by older age, higher proportions of females, absence of a marital partner, and lower levels of education, household income, health literacy, and social support compared to non-frail older adults. Furthermore, the rate of smartphone ownership among frail older adults (30.5%) was less than half that of non-frail older adults (62.4%).

**Table 1:** Characteristics of the study participants (*N* = 2,469)

Variables		Total (N = 2,469)	Frail (N = 269)	Non-frail (N = 2,200)
		Mean (SD) or n (%)	Mean (SD) or n (%)	Mean (SD) or n (%)
Age		77.8 (3.8)	78.2 (3.7)	75.5 (3.8)
	70–74 years	598 (24.2)	25 (9.3)	573 (26.1)
	75–79 years	1,029 (41.7)	86 (32.0)	943 (41.9)
	≥ 80 years	842 (34.1)	158 (58.7)	684 (31.1)
Gender				
	Male	1,175 (47.6)	109 (40.5)	1,066 (48.5)
	Female	1,294 (52.4)	160 (59.5)	1,134 (51.5)
Marital status				
	Married	1,627 (65.9)	154 (57.3)	1,473 (66.9)
	Others	842 (34.1)	115 (42.7)	727 (33.1)
Education				
	< Elementary school	487 (19.7)	100 (37.2)	387 (17.6)
	Elementary school	668 (27.1)	77 (28.6)	591 (26.9)
	≥ Middle school	1,314 (53.2)	92 (34.2)	1,222 (55.5)
Monthly household income				
	< 1 million KRW	1,060 (42.9)	169 (62.8)	891 (40.5)
	1 ~ 2 million KRW	569(23.1)	54 (20.1)	515 (23.4)
	> 2 million KRW	773 (31.3)	37 (13.8)	736 (33.4)
	Don’t know	67 (2.7)	9 (3.4)	58 (2.6)
Smartphone ownership				
	No	1,015 (41.1)	187 (69.5)	828 (37.6)
	Yes	1,454 (58.9)	82 (30.5)	1,372 (62.4)
Health literacy (range 0–12)		7.8 (3.0)	5.7 (2.8)	8.0 (3.0)
Social support (range 8–34)		28.0 (6.9)	27.2 (7.6)	28.1 (6.9)

Note. SD = standard deviation

### Correlation between smartphone ownership, health literacy, social support, and frailty

Table 2 displays the coefficients and *p*-values for the correlation among study variables. Frailty showed negative correlations with smartphone ownership (*phi* = − 0.202, *p =* 0.041), health literacy (*pbis* = − 0.231, *p* < 0.001), and social support (*pbis* = − 0.041, *p* = 0.040). Smartphone ownership exhibited positive correlations with health literacy (*pbis* = 0.360, *p* < 0.001) and social support (*pbis* = 0.081, *p* < 0.001).

**Table 2:** Correlations among study variables (*N* = 2,469)

Variables	Frailty	Smartphone ownership	Health literacy
	Coef. (***p***-value)	Coef. (***p***-value)	Coef. (***p***-value)
Smartphone ownership	–0.202 ^a^ (0.041)	-	-
Health literacy	– 0.231 ^b^ (< 0.001)	0.360 ^b^ (< 0.001)	-
Social support	– 0.041 ^b^ (0.040)	.081^b^ (< 0.001)	0.113 ^c^ (< 0.001)

Note. ^a^ Phi correlation coefficient; ^b^ Point-biserial correlation coefficient; ^c^ Pearson’s correlation coefficient

### Testing for mediation effect

We hypothesized that health literacy would mediate the relationship between smartphone ownership and frailty. Model 4 of the PROCESS macro was adopted to test this mediation effect [37]. Smartphone ownership was positively associated with health literacy (*β* = 0.865, *p* < 0.001), which, in turn, showed a negative association with frailty (*β* = −0.178, *p* = 0.001) even after all covariates were controlled (Table 3). Direct (*β* = −0.623, *p* < 0.001) and indirect effects (*β* = −0.154, Boot CI = − 0.222, − 0.096) were both significant, indicating that health literacy partially mediated the relationship between smartphone ownership and frailty.

**Table 3:** Testing the mediation effect in the relationship between smartphone ownership and frailty (*N* = 2,469)

Variables	Model 1 (Health literacy)						Model 2 (Frailty)				
	β	SE			***p***-value		β		SE		***p***-value
Age	– 0.073	0.014			< 0.001		0.145		0.019		< 0.001
Gender	– 0.437	0.122			0.003		0.117		0.169		0.489
Marital status	0.112	0.130			0.392		– 0.165		0.166		0.320
Education	1.462	0.079			< 0.001		– 0.036		0.102		0.726
Household income	0.310	0.064			< 0.001		– 0.160		0.089		0.073
Smartphone ownership	0.865	0.120			< 0.001		– 0.623		0.160		< 0.001
Health literacy	-	-			-		– 0.178		0.028		0.001
R^2^ / McFadden R^2^	0.299						0.141				
F / -2 log likelihood	174.753***						1460.223***				
**Indirect effect of smartphone ownership on frailty**				Effect		Boot SE		Boot LLCI		Boot ULCI	
Health literacy			– 0.154			0.032		– 0.222		– 0.096	

Note. 5,000 numbers of bootstrap samples for percentile bootstrap confidence intervals. β = regression coefficient; SE = standard error;

R^2^ = coefficient of determination; F = F-test values; Boot = bootstrapped; LLCI = low level confidence interval; ULCI = upper level confidence interval

### Testing for moderated mediation effect

**Fig. 2:**
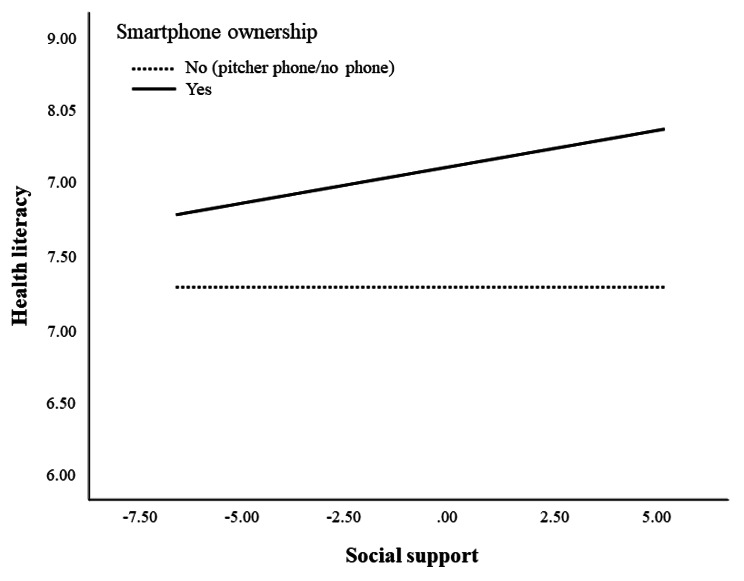
The interaction between social support on health literacy according to smartphone ownership. Note. The figure illustrates the conditional effects of smartphone ownership on frailty at various values of social support. The X-axis represents scores of social support, while the Y-axis indicates health literacy scores. Social support is represented as a mean-centered score. As the solid line displays, an increased health literacy is observed with the risk in social support among smartphone owners, potentially leading to an increased indirect effect of smartphone ownership on frailty.

Regarding hypothesis 3, we supposed that social support would moderate the relationship between smartphone ownership and frailty. Table 4 shows the statistically significant index of moderated mediation (*β* = −0.010, Boot CI = − 0.016, − 0.004), indicating the strengthened indirect effect of smartphone ownership on frailty through health literacy as social support increased. We estimated the conditional effect of smartphone ownership on health literacy at various social support levels to illustrate the moderating role of social support. Health literacy increased among smartphone owners with higher social support levels, and this effect was significant within the range of mean score $\pm$ 1 SD of social support, as shown in Fig. 2. Additionally, the Jonhson-Neyman technique revealed a significant conditional effect at social support levels of > 18.5 points (refer to Supplementary Table 1). However, social support had no significant influence on the increment of health literacy for non-smartphone owners (*β* = −0.002, *p* = 0.882; see Table 4).

**Table 4:** Testing the moderated mediation effect in the relationship between smartphone ownership and frailty (*N* = 2,469)

**Direct relationships**	β		SE		*p*-value	
Smartphone ownership → Health literacy	0.863		0.119		< 0.001	
Social support → Health literacy	– 0.002		0.011		0.882	
Smartphone ownership $\times$ Social Support → Health literacy	0.053		0.015		0.003	
Health literacy → Frailty	– 0.178		0.028		< 0.001	
Smartphone ownership → Frailty	– 0.623		0.160		0.001	
**Indirect relationship**	Effect	Boot SE		Boot LLCI		Boot ULCI
Smartphone ownership → Health literacy → Frailty	– 0.154	0.033		– 0.224		– 0.096
**Moderated Indirect Relationships**	Effect	Boot SE		Boot LLCI		Boot ULCI
Low social support (21.093)	– 0.088	0.031		– 0.154		– 0.033
Mean social support (28.041)	– 0.154	0.032		– 0.223		– 0.096
High social support (34.000)	– 0.210	0.043		– 0.302		– 0.134
**Index of moderated mediation**	– 0.010	0.003		– 0.016		– 0.004

Note. All analyses were controlled for age, gender, marital status, educational level, and monthly household incomes. 5,000 numbers of bootstrap samples for percentile bootstrap confidence intervals. β = regression coefficient; SE = standard errors; Boot = bootstrapped; LLCI = lower level confidence interval; ULCI = upper level confidence interval. In moderated indirect relationship, low social support indicates 1 standard deviation below the mean score and high social support indicates 1 standard above the mean score of social support among participants

## Discussion

To our best knowledge, this is the first study to investigate the relationship between smartphone ownership and frailty among community-dwelling older adults. Our findings indicated that health literacy played a mediating role in the association between smartphone ownership and frailty. Moreover, social support acted as a moderator, thereby enhancing the indirect effect of smartphone ownership on frailty through health literacy. Specifically, the beneficial effect of smartphone ownership on frailty was amplified with higher social support levels.

Frailty has been recognized as a significant contributor to adverse health outcomes in older adults [11]. Understanding the underlying mechanisms that cause frailty in the older population is crucial to develop effective prevention and intervention strategies. Previous research has emphasized the multidimensional approach to frailty prevention, including enhancement of physical activities, nutrition, mental health, and cognitive functions [39]. However, this study highlights the impact of digital device utilization by revealing that older adults who own smartphones are less likely to be frail compared to those who do not own smartphones. This finding offers empirical evidence that smartphone ownership among older adults can be a viable solution to prevent the development of frailty.

Health literacy mediated the relationship between smartphone ownership and frailty. The first part of the mediation pathway indicated that smartphone owners exhibited higher levels of health literacy compared to non-smartphone owners. Although a lack of research specifically investigated the association between smartphone ownership and health literacy, our finding aligns with previous studies reporting a significant relationship between smartphone ownership, internet access, and health literacy [14, 15]. Using a smartphone in older adults is closely linked to internet literacy [40], implicating increased accessibility to online health information. A population-based study conducted in the United States revealed that smartphone ownership was associated with greater confidence in obtaining health information when needed (odds ratio [OR] = 5.63, 95% CI = 1.05–30.23) [14]. Similarly, a national survey in Turkey reported that using smartphones as a source of health information was associated with increased general health literacy (β = 1.974, *p* < 0.01), disease prevention and health promotion-related health literacy (β = 2.535, *p* < 0.001), and healthcare-related health literacy (β = 1.428, *p* < 0.01) [15].

Although this study could not investigate the use of health-related mobile applications among smartphone users due to data constraints, the utilization of these applications can elucidate how smartphone ownership enhances health literacy among older adults. According to a national survey, about half of the Korean older population was capable of installing and using the necessary mobile applications, and this percentage continues to increase with the growing smartphone penetration rate [5]. This increased use of mobile applications has been associated with higher health literacy, particularly in the domains of information accessibility, understanding, appraisal, and practice [41]. Furthermore, mobile health applications can empower individuals by providing features that enhance interactive communication with health professionals and facilitate better understanding of medical information [13].

The second part of the mediation pathway revealed a negative association between health literacy and frailty, which is consistent with previous studies showing health literacy as a significant predictor of frailty [18, 20].

In addition, the direct effect of smartphone ownership on frailty was found to be significant. This suggests the presence of other pathways linking smartphone ownership and frailty that were not explored in this study. For example, smartphone ownership may reduce the risk of frailty through factors such as reduced depression, enhanced social activities, and promoted life satisfaction [9, 10, 39]. Moreover, regular engagement with touchscreen devices may contribute to cognitive performance in older adults, positively impacting frailty prevention [42]. However, there are competing viewpoints suggesting that the use of digital devices may lead to more cognitive concerns by promoting distractibility and lowering memory tasks [43]. Given that 19.4% of males and 22.6% of females in the same population were cognitively impaired [44], future studies should consider the impact of smartphone ownership based on cognitive functions. To uncover underlying mechanisms and provide a more nuanced explanation, it is recommended to explore how smartphone ownership could promote health literacy and lower frailty, considering various factors mentioned above.

Additionally, this study revealed that social support plays a moderating role in the relationship between smartphone ownership and frailty through health literacy. Social support strengthened the association between smartphone ownership and health literacy. Specifically, health literacy was greater when social support increased among smartphone owners. Conversely, social support did not affect the health literacy of older adults without smartphones (Fig. 2). These results are consistent with previous studies that emphasized the positive impact of social support on smartphone usability and online health information-seeking behavior among older adults [23–25, 45, 46]. Lacking social support (e.g., informational, organizational, instrumental, intergenerational, peer support) was identified as a barrier for older adults to engage in navigating online health information [45]. Additionally, social support for older adults promoted health-related online activity by enhancing their empowerment [46]. Our findings revealed a reinforced beneficial effect of smartphone ownership on frailty when older adults had better social support. This result supports previous findings that perceived social support from family or community was associated with low prevalence and incidence of frailty [27, 47, 48].

The present study provides evidence that smartphone ownership and social support levels can generate disparities in health literacy, thereby contributing to health inequalities among community-dwelling older populations. Health professionals and policymakers should integrate health literacy and social support as core components in strategies aimed at preventing and addressing frailty in older adults, particularly when designing interventions that leverage innovative digital technologies. Involving families and neighbors in health literacy education can be valuable for older adults who own smartphones and have sufficient social support to ensure sustainable support in preventing frailty.

Recent studies have reported the effectiveness of interventions using digital technologies in alleviating health disparities among diverse race/ethnicity groups, generations, socioeconomic statuses, and geographical areas [49, 50]. In particular, Korean public health centers have implemented a healthcare program targeting older adults in remote areas, combining smartphone applications and AI technologies to provide non-face-to-face consultations and health education. The initiative has proved effective in preventing frailty, improving self-care abilities, and ultimately reducing health disparities between urban and rural areas, where healthcare service access may be limited [51]. However, equal access to technology-based services depending on smartphone ownership remains lacking, and the lack of proficiency in using these technologies presents another equity problem [13]. Therefore, health professionals and policymakers should advocate for digitally-underprivileged older adults to promote their ability to use technologies for health [6]. Continuing traditional health education programs should be implemented to help older adults without smartphones promote their health literacy.

This study has several limitations. First, due to using secondary data, we assumed that smartphone ownership implies smartphone use among older adults. However, some people own smartphones but may struggle to master their use. Therefore, future studies should assess proficiency in smartphones use, considering age-related functional decline, such as visual and cognitive impairment, to gain a more comprehensive understanding of the relationship between smartphones, health literacy, and frailty in older adults [5, 52]. Additionally, it is necessary to assess whether smartphone owners explored online information and downloaded applications to support their health-related decision or obtained health information from other traditional sources (e.g., television, families or friends) to verify our hypothetical explanations. Second, the measurement of health literacy relied on self-reported, simplistic items assessing the ability to understand health information. However, the concept of health literacy has evolved to encompass digital health literacy. Therefore, adopting a broader understanding of health literacy is recommended to investigate its role in the association of smartphone ownership with frailty. Third, participants in the study were aged ≥ 70 years, who had lower smartphone ownership compared to those in their 60s. Due to the age characteristics of participants, biased results may be possible, warranting caution when generalizing findings to younger older adults aged < 70 years. Fourth, despite controlling for sociodemographic factors as covariates, there are limitations regarding unaddressed confounding factors, including health behaviors (e.g., alcohol consumption, smoking) and medical conditions (e.g., chronic diseases). Furthermore, considering the controversial correlation between cognitive function and smartphone use in older adults, there is a need for in-depth exploration of the mechanism across various levels of cognitive function. Lastly, the cross-sectional nature of the study prevents the establishment of causality, representing another inherent limitation. However, this study is meaningful as it unveils valuable findings by revealing a substantial association between smartphone ownership and frailty while utilizing nationwide data from older adults in Korea.

## Conclusions

The present study addressed the association between smartphone ownership and frailness among community-dwelling older adults. This study revealed that smartphone ownership can decrease frailty. Furthermore, this effect was mediated by health literacy and moderated by social support. These findings highlight the role of health literacy in frailty prevention. Moreover, enhancing social support for older adults with smartphones can help reduce the risk of frailty by improving their health literacy. Additional efforts should be made for non-smartphone owners to improve their health literacy as well as reduce adverse health outcomes derived from the digital divide.

### Electronic supplementary material

Below is the link to the electronic supplementary material.

Supplementary Material 1

## Data Availability

The dataset used and analyzed during the current study are available in the KFACS website, https://www.kfacs.kr/html/.
