# Supplementary material for: The roles of health literacy and social support in the association between smartphone ownership and frailty in older adults: a moderated mediation model
Source: BMC Public Health. 2024 Apr 17;24:1064. doi: 10.1186/s12889-024-18163-z (PMC11037091; doi:10.1186/s12889-024-18163-z)
Supplement: Supplementary file 1 — Supplementary Material 1 [file 12889_2024_18163_MOESM1_ESM.docx]

**Supplementary Table 1.** The moderator values defining the John-Neyman significance region and conditional effect on smartphone ownership at values of social support.

| Social support | Effect | SE | t | *P*-value | LLCI | ULCI |
| --- | --- | --- | --- | --- | --- | --- |
| 8.000 | – .203 | .316 | – .644 | .520 | – .832 | .416 |
| 9.300 | – .134 | .298 | – .450 | .653 | – .718 | .450 |
| 10.600 | – .065 | .280 | – .232 | .817 | – .615 | .485 |
| 11.900 | .004 | .263 | .016 | .987 | – .512 | .520 |
| 13.200 | .073 | .246 | .298 | .766 | – .409 | .556 |
| 14.500 | .143 | .229 | .622 | .534 | – .307 | .592 |
| 15.800 | .212 | .213 | .994 | .350 | – .206 | .629 |
| 17.100 | .281 | .197 | 1.423 | .155 | – .106 | .668 |
| 18.400 | .350 | .182 | 1.920 | .055 | – .008 | .707 |
| **18.499** | **.355** | **.181** | **1.961** | **.050** | **.000** | **.711** |
| 19.700 | .419 | .168 | 2.493 | .013 | .089 | .749 |
| 21.000 | .488 | .155 | 3.148 | .002 | .184 | .792 |
| 22.300 | .557 | .144 | 3.884 | .001 | .276 | .839 |
| 23.600 | .627 | .134 | 4.687 | .000 | .364 | .889 |
| 24.900 | .696 | .126 | 5.517 | .000 | .448 | .943 |
| 26.200 | .765 | .121 | 6.315 | .000 | .527 | 1.002 |
| 27.500 | .834 | .119 | 7.002 | .000 | .600 | 1.068 |
| 28.800 | .903 | .120 | 7.513 | .000 | .667 | 1.139 |
| 30.100 | .972 | .124 | 7.821 | .000 | .729 | 1.216 |
| 31.400 | 1.041 | .131 | 7.939 | .000 | .784 | 1.299 |
| 32.700 | 1.111 | .140 | 7.911 | .000 | .835 | 1.386 |
| 34.000 | 1.180 | .152 | 7.784 | .000 | .883 | 1.477 |
| Value = 18.499, % below = 13.771, % above = 86.229 | | | | | | |

*Note.* SE = standard effort; t = value of T statistics; LLCI = lower level confidence interval; ULCI = upper level confidence interval
